# How Years of Sport Training Influence the Level of Moral Competences of Physical Education and Sport Students

**DOI:** 10.1155/2019/4313451

**Published:** 2019-07-11

**Authors:** Małgorzata Bronikowska, Agata Korcz, Jana Krzysztoszek, Michał Bronikowski

**Affiliations:** ^1^Laboratory of Ethnology of Sport, Poznan University of Physical Education, Krolowej Jadwigi 27/39, 61-871 Poznan, Poland; ^2^Department of Didactics of Physical Activity, Poznan University of Physical Education, Krolowej Jadwigi 27/39, 61-871 Poznan, Poland

## Abstract

**Background:**

The study purpose was to investigate the level of moral competences and prosocial behaviours in a physical activity (PA) context and differences between first year freshman students enrolled in both the physical education and sport coaching majors.

**Methods:**

We draw on data from students of the Faculties of Physical Education (109) and Sport (94) at Poznan University of Physical Education. For measurement, Lind's Moral Competence Test was used. Comparative analysis of differences between the groups was undertaken with the use of Mann-Whitney* U* test and Kruskal-Wallis nonparametric analysis of variance was used for further analysis.

**Results:**

The results show that there were no significant differences between the examined students in majority of the characteristics. Students comply with the recommended levels of physical activity, but 70% present low level of moral competences. Parents were the most influential factors in moral development across levels of competences and genders. More years of sports training showed a positive relationship with moral development (only in the group of male students with high level of moral competency).

**Conclusions:**

Based on the findings, it is recommended to consider the changes in physical education and sport students education training programmes which should include more critical reflection and life-long learning competences.

## 1. Introduction

Physical inactivity appears to be one of the major public health concerns, specifically in young people. The causes of such almost epidemiological situation have been supported with findings and discussed in numerous studies [1]. Social influence sources (parents, peers, and teachers) determine the level of involvement in PA [2, 3]. Lack of youth's interest in PA may be actually rooted in their first educational/sporting experiences and may be connected with the “role modeling” processes played by physical education (PE) teachers and youth sport coaches. PA recommendation and health-related criteria of a physically educated and active person are often provided by national and international health-related boards (like WHO), but are we not missing a discussion on the personal or moral criteria for those who are introducing children to PA? What moral qualities should such educators present themselves? They bear tremendous responsibilities as they play key role in inspiring “young souls” to sports and for the active life style along the lifespan.

Sport used to be one of the best ways for the sound development of a young person, but nowadays it loses its educational value. Better understanding of who, in terms of moral competences, the trainees introducing PA to children are may help prevent the descend of educational sporting experience, specifically when the moral quality of the future educators is taken into consideration.

Promotion of positive human values and behaviours that matter for the moral development of a young person is part of regular teaching conduct in PE. This should also be applied to sports education, but it has been questioned whether it can be taught by a sports coach, as the two qualifications pathways (PE teaching and sports training) differ in aims, methods, strategies, and contents, as well as in the type of participants and their motivation. Potential problems may stem from the fact that sport coaches often lack formal training in teaching social and moral development. What influences the level of moral competences in preservice educators in PA (namely, PE teachers and coaches) is not known and became a central point in this research.

Teachers play an important modelling role [4]. Sanderse [5] indicates that role modelling is rarely used as an explicit teaching method, and only a very small percentage of pupils consider teachers as role models. Chen and Ennis [6] claim that PE teachers present the “disciplinary mastery” orientation focusing on developing performance proficiency in sport skills and understanding of performance-related knowledge, whereas Green [7] sees orientation of PE teachers as an “amalgam” of teachers' subconscious predispositions and practical situations in which those teachers found themselves. However, in case of the potential influences teachers of all subjects play on pupils' moral development, much depends on PE teachers and school sport coaches, as they have a wider range of means and ways to positively affect pupils' sound development and engagement in PA or quite opposite discourage them from PA on the false idea that sport is a separate sphere of human behaviours. A central finding of Šukys et al. [8] shows that the personal role of sport coaches in moral education, encompassing not only professional knowledge but also moral competences in young athletes, is of great importance and influences one's moral development greatly. On the other side, moral education has more to do with the character and virtues of the PE teachers rather than any particular didactic skills they possess [9]. Jones [10] claims that virtues should be embodied in the character of a teacher so they can be applied directly in the teaching process, rather than post hoc or hypothetically. It seems important to find out when they (teachers/coaches) gain those virtues, whether it is during their schooling/sporting education or during their study years.

Theodoulides [11] indicates that some PE teachers merit the fittest pupils and perform only instrumental actions that emphasize behaviours aimed at winning, sometimes leading to unsporting/unfair play. It is hard to believe that those educators do not realize that such inappropriate behaviours may actually discourage from PA those less fit children, perhaps with smaller movement competency but a greater sense of fairness, for example. Can this kind of “role modelling” be motivating for young pupils and become an inspiration for a long-term commitment to sport, or at least leisure time PA?

It is quite possible that pupils internalize those low moral standards and repeat them not just during their years of involvement in school sports as young athletes, but later, when they become parents. They start introducing their own children to PA forcing ‘winning at all cost' and ‘survival of the toughest' philosophy, the same they had learned in their school years. Shouldn't school PE and sport be about stimulating development of the best qualities in a child, no matter what level of fitness they present? Every child deserves such a chance.

In the early phases of life development of a young person, introduction to PA comes either through school or a sport club. According to education policies and curriculum documents of most European countries, all school subjects are responsible and should equip a pupil with moral competences. However, educational programmes revile the idea that school subjects undertaking some aspects of moral education (e.g., ethics, religious education, or mother tongue) are unilaterally geared to raising the knowledge of moral conceptions, which are easy to be tested in students, but they may have little in common with peoples' behaviour towards each other [12]. Perhaps we need to look at the qualities of the educators then.

There are certain differences in qualifications needed for teaching and coaching. In Poland, an undergraduate or master's degree is required to teach PE in schools. To become a coach one can either obtain a degree or gain qualifications from a relevant sport federation; however, such a qualification is not sufficient to teach PE in schools. And, for example, in Sweden [13], teaching requires a valid teaching certificate with relevant subject knowledge, but in the case of school sports, when no qualified PE teachers are available, other qualifications can be recognised. As Ferry [13] reports, schools frequently employ sport coaches instead of PE teachers. Shields and Bredermeier [14] observed a fact of more years in sport-related backgrounds in sport coaches than among PE teachers, which may be a natural advantage in a job application process. A similar situation has been reported by Blair and Capel [15] in English primary schools, where teaching of PE can be undertaken by coaches holding national governing body awards, even without teaching qualifications. It is also the case in North America [16].

There has been some research on the potentially conflicting overlap of the two roles dealing with PA education, namely, PE teachers and sport coaches. Dodds et al. [17] examined the differences between teacher and coach recruits and made comparisons to other sports-related occupations. They found out that the two groups' most influential factors in terms of the role differed in academic and sporting backgrounds, with teacher/coach recruits participating in sports more and longer during high school and college years than just teacher recruits. Locke and Massengale [18] examined occupational conflicts related to role-overload and found that it is often female teachers/coaches that experience these more intensely. When Bain and Wendt [19] investigated undergraduate PE students' perceptions of the teacher and coach roles, it was noticed that students viewed the abilities for each of the roles as similar. However, the gender of a student played a significant part in a role preference, with male students indicating a greater preference for the coaching role, and this was related to the estimated ability of being a role model possessing very high level of skills in a specific sporting activity. In contrast, students of both genders who preferred the teaching role indicated less experience in sports. Meanwhile, in the context of sport, athletes with strong moral identity reported less frequent antisocial behavior toward their opponents [20, 21]. But in terms of moral competency a study by Walker [22] revealed that there is no relation between gender and moral stage of one's development, and further, Proios and Doganis [23] found that there are no significant differences in moral judgment schemas between genders, and likewise are results from Salvano-Pardieu et al. [24]. The results of the study of Mouratidou and Barkoukis [25] revealed that male secondary education students reported higher scores of negative behaviour towards them performed by a teacher in PE setting, when compared to female peers. This also includes students and their beliefs about gender inequalities (biases) in PE courses.

There is a lack of research concerning the problem of whether social factors (such as religion, school education, peers, parents, and teachers) or sport factors (years of training and type of sport) impact moral competences of future PE and sport specialists. However, more recent research has moved beyond testing moral disengagement which refers to a set of eight cognitive mechanisms that decouple one's internal moral standards from one's actions, facilitating engaging in unethical behaviour without feeling distress towards understanding, when it operates as a mediator and moderator of other relationships [26]. Therefore it would be worth finding out what levels of moral competences are presented by the recruit students of the two professions.

The theoretical framework for the study was based on Kohlberg's cognitive developmental theory of morality [27] and, derived from this work, Horrock's Prosocial Play Behaviour Scale (HPPBS) [28], which includes moral judgement, reasoning, and intentions. As Gibbons and Ebbeck [29] state, moral judgement and intentions are important indicators of one's behaviour at a given moment, whereas reasoning is related to cognitive maturity, which is in line with Kohlberg's theory. Lind's [30, 31] Moral Competence Test (MCT) was used, which assesses moral competences as well as one's preferred attitudes. The MCT scoring system (*C-index*) takes into account the overall pattern of one's moral competency and thus provides a reliable evaluation. A person can score high on the* C-index *only when they show moral consistency in judging of pro and contra arguments in the test, which is a characteristic of those with more developed cognitive structures and the capacity to value opposing viewpoints. A lack of these skills and no moral consistency will automatically lead to scoring low but will not eliminate such a person from a schooling or sporting context of working with children.

The limited literature on moral competences of PE and sport major students underlines the significance of the present study. Professionals working with children and youths in area of PA should present the highest standards themselves first. A study on over 440 PE students from all studying years [32] revealed that almost 80% of them presented a very low level of moral competences and the lowest value on the influence scale was attributed to PE teachers. So we wondered whether it was a problem connected to the study programme or earlier PA experiences, and therefore it was interesting to investigate the situation with the newly recruited first year freshmen, because the students' moral consistency might be a crucial factor for the quality of teaching moral education through PE and school sport to the youths.

In the abovementioned study [32] also religion did not play a significantly influential factor in the moral development of PE preservice students. Religion and church continue to have a marked significance in Poland. It represents an active force that can assume both a unifying and divisive character. On the other hand, it is undisputed that the observable religious changes in Poland are characterised by general dechurchification which means most of the Polish population leaves the traditional upbringing pattern for the secularization [33]. This is quite interesting observation in Poland, a country known for its strong catholic roots.

Based on the above, the present study was aimed at finding the estimates of levels of moral competences, prosocial play behaviours in PA context, and potential differences between potential PE teachers and sport coaches. A secondary purpose of the study was to investigate the context of gender differences between preservice PE teachers and coaches. In order to assess the level of moral competency at the beginning of the professional career and to eliminate a potentially strongly influential role of a study programme, a comparative analysis between the first year recruit students of abovementioned professions was undertaken.

## 2. Method

### 2.1. Research Design

A cross-sectional studies design was used. This type of study measures the outcome and the exposures in the study participants; at the same time the participants are selected based on the inclusion and exclusion criteria set for the study. Once the participants have been selected, the investigator follows the study to assess the exposure and the outcomes [34]. It was aimed at defining the current relationships between the examined concepts in this study as well.

### 2.2. Research Sample

To determine the sample size reasonably reflecting the target population, a sample size calculator was used [35]. Concerning the total population of the first year students of physical education major (143) and sport major (108) at the Poznan University of Physical Education, the minimal sample size should be made of 104 and 84 students, respectively, in each of the majors. The study data was collected in 2017 including 109 (60 male/49 female) physical education major students, which represented 76.2% of the first year total, and 94 (59 male/35 female) sport major students, which represented 87% of the first year total at the Poznan University of Physical Education. The mean age of the examined sample was 19.67±1.35 years for male students and 19.33±0.58 years for female students.

### 2.3. Research Instruments and Procedures

For the purpose of the study, a Polish validated and certified version of MCT was used [36] based on Moral Competence Test [30], formerly known as the Moral Judgment Test. According to the theory, moral competence is defined as the ability to cope with problems and to resolve conflicts on the basis of inner moral principles, not on the basis of external social expectations [31]. All responders were confronted with two different hypothetical moral dilemmas and were requested to rate their views on a 9-point Likert-type scale, from -4 = totally disagree to +4 = totally agree. Every story had 12 statements (6 in favour and 6 against the proposed behaviour). The summarized score (*C-index*) was computed and ranges from 1 to 100. The final score refers to the individual's ability to assess an argument based on their moral quality: it measures the degree to which a person allows their personal judgments and reasoning to be affected by moral concerns or principles rather than their personal opinions and constructions.* C-index* below 9 points was considered as very low, below 19 as low, between 19 and 29 as medium, above 29 to 39 as high, and above 39 as very high. For the purpose of the study, students were categorized into three levels of moral competences: low and very low (≤ 19), medium (19-29), and high and very high (≥ 29).

In this study, the role of different social factors influencing moral competences was evaluated with the use of an 8-item scale (religion, school education, parents, PE teacher, sport coach, peers, media, and studies) developed specifically for the purpose of the study by the authors themselves. The scale consisted of a question ‘What influences your moral competences?' A Likert scale was used with scores ranging from 1 = not at all to 5 = very much. Test-retest reliability values (with a two-week interval) for the same items ranged between 0.66 and 0.88.

To measure the declared prosocial behaviours of examined students, “My Physical Education Class” [37], a questionnaire based on Horrocks [28], was used. Prosocial Play Behaviour Inventory (HPPBI) was used. The questionnaire measures moral judgement, reasoning, and intentions of the participants through a self-rating system. Judgement and intentions are scored on 3-point and reasons on 5-point Likert scale ranging from 1 = not at all to 5 = very much. Horrocks [28] provided content and construct validation, as well as good reliability, for the HPPBI. The internal consistency for the moral behavior scale was 0.75.

The level of PA was determined with a scale constructed by Prochaska, Salis, and Long [38] and called Physical Activity Screening Measure. It assesses the average number of days per week with at least 60 minutes spent undertaking various forms of PA during which, in the participants' subjective opinion, their heart rate increased and they experienced a feeling of shortness of breath (higher breathing frequency). Examined students were asked two questions: P1: Over the past 7 days, on how many days were you physically active for a total of at least 60 minutes per day? P2: Over a typical or usual week, on how many days are you physically active for a total of at least 60 minutes per day? This formed a moderate-to-vigorous PA index (MVPA). The MVPA index was calculated according to the original formula presented by Prochaska et al. (2001): MVPA = (P1 + P2)/2, where MVPA = PA index; P1 = number of physically active days during the past 7 days; P2 = number of physically active days during a typical (usual) week.

As this study involves human data, authors declare that the investigations were carried out following the rules of the Declaration of Helsinki of 1975 [39], revised in 2013. According to point 23 of this declaration, the study protocol was approved by the Local Bioethics Committee of University of Medical Sciences in Poznan (decision no. 908/16) before undertaking the research.

For the students' convenience, the information about the anonymous and voluntary nature of their participation was pronounced, the study records were kept confidential, and their individual contributions were unidentifiable in the final report.

### 2.4. Data Analysis

Due to the lack of normal distribution, comparative analysis of differences between the groups (PE/sport students) was undertaken with the use of Mann-Whitney* U* test. As there were no major differences between the students of both faculties (the only difference among all variables was noticed in items* school*, p=0.04, and* years of professional training*, p=0.001), the next step was dividing the whole group into female and male subgroups within the three levels of moral competences and analyzing differences between the genders with a subsequent Mann-Whitney* U* test. To analyze the potential role of involvement in sport (years of training) on moral competences (low, medium, and high levels) in male and female students a Kruskal-Wallis nonparametric analysis of variance was used. To identify subsets of most important variables and to evaluate the order of importance, this was followed by a stepwise regression method for each of the subgroups (female/male and three levels of moral competences). Significance was set at p ≤ 0.05. Statistical analysis was carried out using Statistica 13.0 software.

## 3. Results

A comparative analysis of whole group scores between PE and sport students showed no statistically significant differences in most of the examined variables (except for* religion* in favour of PE students and* school education* in favour of sport students). Since there were no statistically significant differences between the groups of students from the two faculties they have been grouped together and analysed for differences between genders within three levels of moral competences. The proportions in the three levels were as follows: group of low moral competences: 70% of the total sample (male students 42%, female 27%), medium level of moral competences: 18.7% from the sample (9.3% of students of each gender), and high level of moral competences: 11.3% of the total sample (6.4% female students, 4.9% male).

In the group of low moral competences, significant differences (Table 1) between genders were found in the role of* sport coach* (p=0.03 in favour of male students) and* judgement *(p=0.01 in favour of female students). No other variables showed any differences between the genders at this level of moral competences. There were more cases of statically significant differences between male and female students in the group with a medium level of moral competences and interestingly in all the cases female students indicated higher mean values as compared to male PE and sport students. Higher values were assigned by female students to the role of* school education *(p=0.05),* PE teacher *(p=0.03),* peers *(p=0.03),* media *(p=0.03), and s*tudies *p=0.01). Finally, among students presenting the highest level of moral competences significant differences were observed in factors* religion *(p=0.01),* parents *(p=0.04), and* sport coach* (p=0.03) all in favour of male students, with girls gaining higher mean value only in* judgement* (p=0.05). Worth noting is the relatively low value attributed to the significance and role of* PE teacher* in moral development in both genders and across the levels of moral competences (lower than the role of s*port coach*). Another point of interest is the high value attributed to the role of* parents* in all the groups at each level of moral competences. Religion was significant for male students from the group of the highest level of moral competence. There were no significant differences between the genders in C-index within each of the levels of moral competences.

In terms of PA-related factors (Table 2), there was a difference in MVPA levels noticed between female and male students, but only in the group with the highest level of moral competences and in favour of males who were more active (0.03) meeting recommendation by WHO of the amount of weekly PA (5 days a week, 60 minutes of MVPA). One particularly striking result was the difference in the years of sport professional training between male and female students (p=0.01) from the group of the highest moral competences, with males indicating 3 times longer (6.9 years) sporting experience than female students, who declared shorter experience of professional sport training (1.9 years on average), and also lower than in the other groups and levels of moral competences. Female students from that group also indicated lower experience in amateur sport training (2.2 years). However, in this case it was comparable to male students (1.6 years), but it was still lower than in the other two groups (medium and low level of moral competences).

In male students Kruskal-Wallis analysis of variances in levels of moral competence with* years of amateur training* did not show any significance (H (2, N=119)=2.07; p=.3540), whereas in case of* years of professional training* it was showing some tendency (H (2, N=119)=5.25; p=.0724). In female students levels of moral competences indicated no differentiation neither* in years of amateur training* (H (2, N=84)=.52; p=.7685) nor* in years of professional training* (H (2, N=84)=1.55; p=.4593).

**Figure 1:**
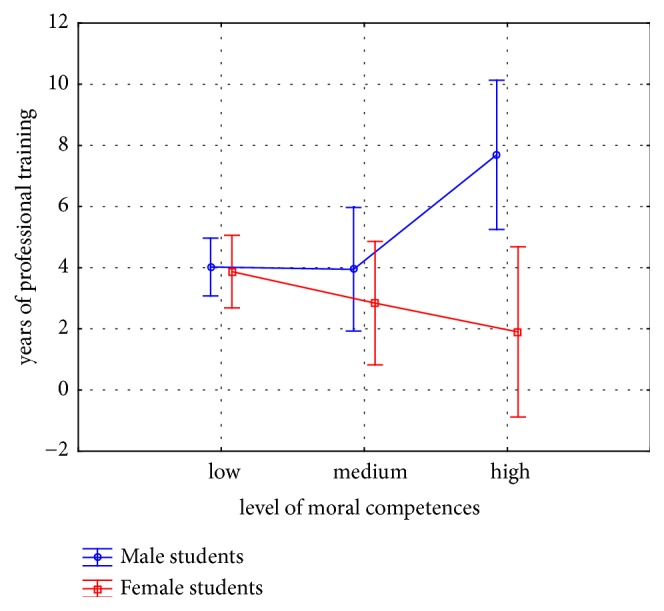
Differences in moral competences with years of professional training in PE/sport students.

Further analysis with Mann-Whitney* U* (Figure 1) testing for differences between genders at each level of moral competences (accordingly in* years of amateur* and in* years of professional training*) indicated that only in case of male students longer involvement in professional sport was associated with higher moral competency, whereas for female students years of training did not play such a role. This was statistically significant (U=28.0; z=2.26; p=0.01). No such differences between male and female students were observed in case of years of participation in amateur sports.

In the six regression analyses (for each level of moral competences, for male and female students separately) the results indicated significant relationships between moral competences (*C-index*) and the selected variables. In the male group of students with the lowest level of moral competences (F(5, 81)= 3.78, p=0.003), a negative relationship was found with* intentions *(*β* = -0.265, p=0.011) and a positive one with* years of professional training *(*β* = 0.218, p = 0.032), which explained 10% of the variance. In the male group with the highest level of moral competences (F(2, 13)=3.78, p=0.053), only the factor of* judgment* was included in the regression model (*β* = -0.712, p=0.020) in the negative relationship, which explained 31% of the variance. A similar situation was also observed in female students with the highest level of moral competences regression model (F(2, 10)=4.29, p=0.025), where* judgment* factor, also with a negative relationship (*β* = -0.790, p=0.021), came into the model, which explained 42% of the variance.

## 4. Discussion

Teaching and coaching qualifications in Poland are gained through training at universities of applied sport sciences, so potential study candidates entering the studies have their minds already set on that target and are more or less aware of the pathways of future careers. But their responsibilities reach much further, as they are going to educate young generations to life-time long PA, one of the most crucial health-related factors in the quality of life.

The scores on MVPA levels of preservice professionals (PE and sport students) indicated its high compliance with recommended standards of weekly PA levels (according to WHO, five days a week with 60 minutes of moderate-to-vigorous PA). In all groups of levels of moral competences the average levels of MVPA varied from 4.6 to 5.6 days/week in both male and female students. The only lower level of MVPA (4.3 days/week) was noticed in group of female students with the highest level of moral competences, but even that is still a reasonable level. So it seems that the freshman recruits students of physical education and sport majors do adjust the levels of PA to the social expectations as role models and to the recommended health standards. However, the question arises: what is the situation with their other competences?

In our research, the majority of examined PE and sport preservice students were classified as presenting low level of moral competences. Additionally, there were no significant differences between first year PE and sport students in most of the variables, which may reflect recruiting profile of the two faculties—young people with some earlier experiences and involvement in various forms of PA. The role of PE teacher is stereotypically interchangeably confused with the role of school sport coach. This is no wonder as it is very often the case that the same person plays the role of a PE teacher and a sport coach in extracurricular physical activities, thus using the same methods, teaching styles, and range of tasks in both roles. This also has to do with the approach to the teaching programmes in school PE. Own experience seems to play fundamental role in the development of teaching/coaching expectations, practices, and moral attitudes.

Research by Henkel and Earls [40] revealed that low level of moral competences of physical educators is unsurprising and it seems that young PE teachers are less developed in moral reasoning capacities than most peers of a similar age and with similar educational backgrounds. Findings of Cummings et al. [41] indicate that it is a wider problem—moral reasoning levels of preservice and in-service teachers are generally relatively low. The importance of highlighting the moral aspects in training for the teaching profession and in teacher education programmes has been raised in other previous studies [4, 42]. In Lampe's study [43] it has been suggested that student-teachers' generally low levels of moral reasoning may be explained by the tendency for teacher education curricula to focus mainly on knowledge and their subsequent neglect of ethical issues. In the case of PE students (especially during the first year of their studies), the problem might also be a misconception in understanding the modern paradigm of PE, which may be still anchored in a traditional physical sport-centred approach as much more present than a holistic one, enhancing social attitudes, moral development, and life-long learning skills in PA. Therefore, the most worrying finding in our study is the fact that the biggest in number group of students of both majors presented the lowest level of moral competences.

In light of public health problems (physical inactivity, obesity, hypertension, and cardiovascular diseases) and moral concerns modern sport is facing, the education of children in PA requires professionals with the highest moral standards themselves, but, as our findings show, they are scarce. This might be an outcome of how they were educated/trained through school PE/sport experiences earlier on themselves. Shields and Bredemeier [14] in their research on school sport claim that school sport if aimed at ‘winning at all cost' may cause several problems. In examined youth, cheating was approved by 14% of the teenagers, 32% thought it was fine to argue with officials, 22% accepted trash-talking as simply part of the game, 29% approved booing, 12% agreed that faking an injury is acceptable, and 41% accepted flashy, egotistical celebration. Situated within this context, our results may come as a no surprise, but they do call for more training on moral issues for preservice PE teachers and coaches and other PA related professionals.

Interestingly, female students from the groups of low and high levels of moral competences presented significantly higher levels of* judgement *competences in terms of prosocial play behaviours than their male student peers. This might indicate their awareness on distinguishing what is right from what is morally wrong, which perhaps needs to be enhanced with age-appropriate reasoning gained via proper moral education. For example, studies by Shields and Bredemeier [14] revealed no differences in moral reasoning between high school basketball players and nonathletes; and it indicated higher and more mature moral reasoning in females in general, although the sample was small.

In our study, it was the male group of the students with the longest experience in professional sport training who showed the highest level of moral competences. At the same time, among female students from the same level of moral competences, longevity (the shortest of all groups) of involvement in professional sports played no significant role in terms of total moral competence score (*C*-*index*). However, years of sport training in the regression analysis only indicated a positive relationship with the* C*-*index* in the group of male students with the lowest level of moral competences. It seems that the shorter girls are involved in professional sport the better for their moral development. There are some studies that contributed to the empirical evidence demonstrating that sport per se does not build moral character indicating that it happens only under certain circumstances [44, 45]. The explanation of our results might be that the process to the learning of values in that group was not trained appropriately and most probably was lacking guidance by established theories, such as Koh's [46] or Kohlberg's [27] theory, or maybe not trained at all.

More recently Richards and Templin [47] used a multidimensional perspective to show that problem of overlapping of the two professions still exists in terms of PE teaching and coaching. They stressed that it is wrong to assume both roles could be performed by the same person without any problems, which was claimed to the differences in skill requirements and social demands. Although both professions focus on skill instruction in physical activities, in case of sport coaches it is their own experience in sports at a competitive level and familiarity with sporting settings that matter the most, whereas teaching PE involves more pedagogical contents and skills. Vella, Oades, and Crowe [48] signalled that even if coaches have access to formal coach training, coach education programs lack content that is relevant to positive youth development, instead maintaining a focus on technical and tactical skills. Lee [49] sees the distinction between PE, which, he believes, is more about personal development, and sport, which focuses more on excellence in physical skills and competition. And Drewe [50] stresses that PE involves equipping pupils with a wider system of beliefs through the development of cognitive and affective systems. Looking at examined young adolescents (first year recruits of PA-related studies) who have just left school PE system to continue their engagement in becoming PA specialists, the results of this study do not confirm that belief.

Interestingly, in our study the significance of influence on moral development and role of PE teacher was not highly valued by either male or female students in any of the three groups of moral competence levels. It was given less credit in terms of enhancing moral development by, for example, sport coach. This observation can be explained by the ambivalent perception towards PE teachers in Polish society and the relatively low social status of PE as a school subject still perceived more like a sport-training process than PA and health-enhancing, awareness building one. On the other side, the highest merits were ascribed to the role of parents, and this pattern was visible regardless of gender and moral competence level, which sounds optimistic. In a study by Ortiz Barón, Apocada Urquijo, Etxebarria Bilbao et al. [51], it was found that the principal variable in predicting internalized behaviors in girls was maternal affection, whereas for boys it was maternal emotional communication and transmission of values.

Smetana [52] believes morality is constructed from reciprocal social interactions; thus both affective and cognitive components of parents' interactions with their children may facilitate children's moral development. The affective context of the relationship may influence children's motivation to listen to and respond to parents; in addition, affection associated with responses to transgressions can affect children's encoding and remembering of those events. Although moral interactions occur frequently in peer contexts, parents' domain-specific feedback about the nature of children's moral interactions is proposed to provide a cognitive mechanism for facilitating moral development. So the high value accredited by examined PE and sport recruit students to parental influence should be utilized in the future for increasing the awareness of parents and their role in the sound development of their children, also in the context of sport education.

Kohlberg and Candee [53] believed that the more mature one's moral reasoning is, the more reliably reasoning and action would cohere and the more action choices would be morally defensible. Higher stages of moral development allow individuals to form more justice-based orientations which can lead to an impartial and universalizable mode of resolving moral dilemmas. However, this may differ in the field of sport. Shields and Bredemeir [14] argue that the world of sport is an area governed by rules and artificially designed goals, based on hierarchy and well-organized structures, where authority figures and roles are less dominant. According to the authors, it is likely that the values and norms of heteronomous morality will be prominent in an athlete's reasoning in the sport-specific context [14] and this may provide an explanation as to why many athletes use a unique form of “game reasoning” in moral dilemmas in sport. However, as it appears from research among individuals with long sports experience, this characteristic way of moral reasoning may extend to moral dilemmas outside sport as well.

In general, PE teachers as well as coaches can develop youth's values system with success; however delivering such programs is highly dependent on their ability and knowledge [54]. Furthermore, experienced PE teachers and sport coaches recognized the need to have teaching/coaching philosophies that are connected to the mandate of the school context in which they operate [44]. Since the role of religion has been marginalized, at least in Poland and among PE and sport recruit students who have not assigned particularly high notes to the importance of that factor in their moral development and which is not perceived any longer as one of the most fundamental agents of value teaching, the bigger role in this task needs to be taken by the higher education providers. This may be a part of a wider sociopolitical change and an effect of democratic transformation of the last 25 years in Poland. Religion in Poland, once associated with national identity, freedom, and defence of human rights and democracy, has to face and cope with significant political, economic, and social changes, from dualism to pluralism [55]. Now, as Catholic Church in Poland concentrates on providing compulsory religion education to school (taught at every level of education with 2 lessons a week) that could raise some tensions and therefore loosen a bit traditional ties with the Church in young generations. Another issue may be the generation of so-called Millennials. In a study by Stephens [56] on American Millennial generation students, the mean* C-index* value was 17.1 points. A significant correlation between moral judgment competence and parental attachment was observed, indicating a strong fostering role of parents in moral development of their children. But the most crucial time for moral development was when students changed from secondary to academic education as they often disengaged from their parents (or maintained contact via modern media) and want to become autonomous persons.

Thus, it appears clear that universities have to equip preservice PE teachers and sport coaches with skills needed to connect this with the acquisition of values and enhancement of sound development (critical thinking, autonomy, drive for professional development, and health-related awareness). However, some studies [57] suggest that teacher programmes are insufficient in providing preservice teachers with life-long learning competences, especially concerning self-improvement, and teacher candidates are critical of the training program and quality of the academic staff [58]. This needs to be taken into account seriously.

Although the examined sample is quite reasonable, the distribution for the three levels of moral competences was dominated by those with a low level of moral competences, which might be considered as one of the limitations of the study. For this reason, it is recommended to apply the study to different sample groups.

## 5. Conclusion and Recommendations

In this article, we have explored the strength of factors influencing moral competences of PE and sport recruit students. Majority of examined students, studying to become PA professionals, complied in accordance with the recommended 5 times a week with 60 minutes moderate-to-vigorous PA standards. Unfortunately, the majority of the examined first year students presented a low level of moral competence, which should be rather a worrying finding in light of the fact that these students will soon join the education system as either professional PE teachers or sport coaches and be responsible for encouraging children to life-long PA. Additionally, the low value accredited to PE teacher's role in terms of moral development should be a warning sign for the education system. On the other hand, parents were identified as one of the most influential factors. This is optimistic, because it may stimulate the development of more mature moral thought of a young generation through parental support. More years of sports training showed a positive relationship with moral development, but only in the group of male students with the highest level of moral competency. However, the relationship might not be so linear, as we do not know the initial level of moral competences of those students when entering sports training process. It is possible that it was not the sport that kept their moral development at that level, but that the initially high level of moral competences helped them to stay within sport training for that number of years.

Nevertheless, we recommend that future teacher education training programmes should be based on and include more critical reflection and health-aware tasks and life-long learning competences, which are essential for promoting and developing individual moral competences. This may also help in increasing the public health and policy makers' awareness and importance of educational role teachers/coaches play in the overall sound development of youths. Stated simply: PA professionals working with children and youths should present the highest standards themselves first. For future research, it is recommended for the focus to be placed on the level of moral competences of students from other academic teaching professions, which could be examined due to the fact that sound development of a school pupil relies on the whole school education setting and on teachers of all subjects.

## Figures and Tables

**Table 1:** Differences between genders (PE and sport students together) in examined prosocial variables (mean ± SD) within the levels of moral competences.

	Pre-service PE and Sport students								
	Low moral competences		p value	Medium moral competences		p value	High moral competences		p value
Variables	Male	Female		Male	Female		Male	Female	
	n = 87	n = 55		n = 19	n = 19		n = 13	n = 10	
Pro-social factors									

Religion	2.8±1.73	2.8±1.58	0.90	2.4±1.89	3.0±0.94	0.16	3.6±1.19	1.4±1.42	0.01
School edu.	2.6±1.19	2.8±1.21	0.44	2.0±1.31	3.0±1.05	0.05	2.6±1.38	2.2±1.39	0.42
Parents	3.9±1.10	4.0±0.90	0.75	3.4±1.30	3.9±1.17	0.12	4.2±0.83	3.2±1.22	0.04
PE teacher	2.3±1.31	2.1±1.13	0.20	2.0±1.41	2.9±1.12	0.03	1.7±1.30	1.8±1.61	0.89
Sport coach	3.5±1.22	3.1±1.24	0.03	3.5±1.30	3.4±0.83	0.57	3.5±1.26	2.5±0.84	0.03
Peers	2.8±1.22	2.8±1.14	0.93	2.7±1.28	3.4±1.01	0.03	2.7±1.37	2.1±0.73	0.19
Media	1.9±1.26	2.0±1.06	0.59	1.5±1.12	2.4±1.30	0.03	1.5±0.96	1.6±1.34	0.94
Studies	2.6±1.17	2.8±1.06	0.55	2.5±1.12	3.4±0.84	0.01	2.1±1.34	2.7±1.25	0.26
Judgement	2.2±0.27	2.5±0.22	0.01	2.4±0.20	2.4±0.24	0.39	2.5±0.19	2.7±0.20	0.05
Reasoning	3.6±0.62	3.7±0.49	0.35	3.7±0.58	3.9±0.41	0.20	3.6±0.69	3.9±0.79	0.31
Intention	2.4±0.22	2.4±0.23	0.30	2.4±0.24	2.4±0.23	0.97	2.3±0.44	2.5±0.30	0.20
C-index	8.6±5.06	8.7±4.93	0.86	24.1±3.06	24.5±3.09	0.54	37.2±6.98	39.0±6.19	0.43

**Table 2:** Differences between genders (PE and sport students together) in examined PA-related variables (mean ± SD) within the levels of moral competences.

	Pre-service PE and Sport students								
	Low moral competences		p value	Medium moral competences		p value	High moral competences		p value
Variables	Male	Female		Male	Female		Male	Female	
	n = 87	n = 55		n = 19	n = 19		n = 13	n = 10	
PA related factors									

MVPA	4.6±1.62	4.7±1.50	0.91	4.7±1.25	4.7±1.42	0.94	5.6±1.19	4.3±1.37	0.03
Years of amateur training	3.5±3.19	3.4±3.28	0.86	3.4±2.52	2.6±1.50	0.98	1.6±3.56	2.2±1.86	0.34
Years of professional training	4.0±3.37	4.1±3.92	0.92	3.9±2.30	2.9±1.95	0.70	6.9±4.44	1.9±1.87	0.01

## Data Availability

The Statistica 13.0 software data used to support the findings of this study are included within the supplementary information file(s).

## Supplementary Materials

Supplementary Materials The Statistica 13.0 software data used to support the findings of this study.
